# Supplementary material for: The Relationship between Brachionus calyciflorus-Associated Bacterial and Bacterioplankton Communities in a Subtropical Freshwater Lake
Source: Animals (Basel). 2022 Nov 18;12(22):3201. doi: 10.3390/ani12223201 (PMC9686566; doi:10.3390/ani12223201)
Supplement: Supplementary file 1 [file animals-12-03201-s001.zip › Supplementary Figures.pdf]

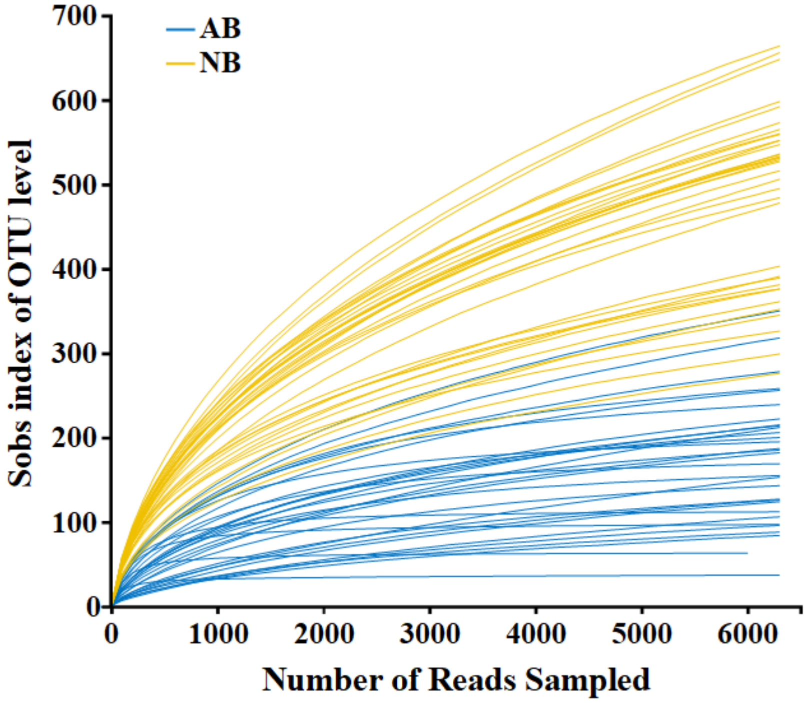

Figure S1. Sparse curve of bacterial community. The abscissa represents the amount of sequencing data randomly selected, and the ordinate represents the number of species observed.

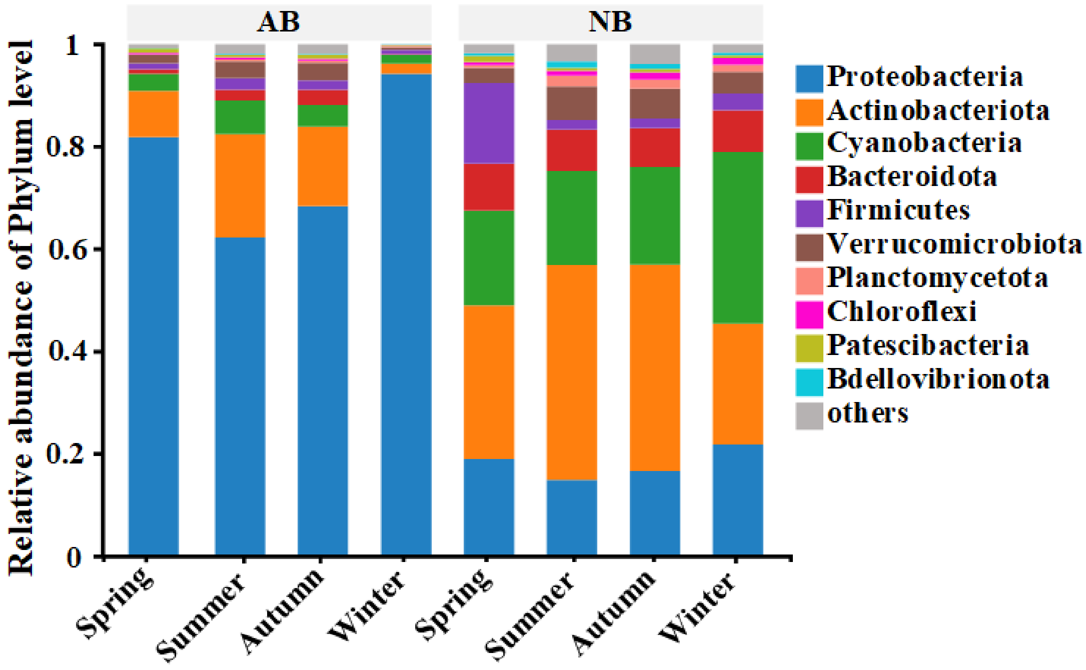

Figure S2. Seasonal variation in the community composition of *B. calyciflorus*-associated bacteria and bacterioplankton at phylum level.
